# Exploring the technological acceptance of a mobile learning tool used in the teaching of an indigenous language

**DOI:** 10.7717/peerj-cs.550

**Published:** 2021-06-03

**Authors:** Santiago Criollo-C, Mayron Lema, Mario Salvador Gonzalez, Angel Jaramillo-Alcázar, Andrea Guerrero-Arias, Sergio Luján-Mora

**Affiliations:** 1 Facultad de Ingeniería y Ciencias Aplicadas, Universidad de Las Américas, Quito, Pichincha, Ecuador; 2 Department EGB/BGU, Jezreel International Christian Academy, Quito, Pichincha, Ecuador; 3 Department of Software and Computing Systems, Universidad de Alicante, Alicante, Spain

**Keywords:** Mobile learning, m-learning, Mobile applications, Mobile devices, Kichwa, Learning, Education, Mobile app, DSR, UTAUT Model

## Abstract

Language is the primordial element for cultural transfer in indigenous communities; if it is not practiced, there is a risk of losing it and with it, a large part of the history of a community. Ecuador is a multicultural and multiethnic country with 18 indigenous peoples. Currently, in this country, some native languages are at risk of disappearing due to factors such as racial discrimination, underestimation of the language, and, above all, the lack of interest and motivation of the new generations to learn this language. Information technologies have made it possible to create mobile applications such as games, dictionaries, and translators that promote the learning of the Kichwa language. However, the acceptance of technology has not been evaluated, nor the intention to involve mobile devices in the process of teaching this language. Subsequently the objective of this work is to explore the acceptance of technology and the use of mobile devices to motivate the learning of the Kichwa language. For this purpose, the mobile application “Otavalo Rimay” was used with several students of a Kichwa language learning center. The methodology used to verify the hypothesis of this work was Design Sciences Research (DSR) together with the theory of acceptance and use of technology (UTAUT). The instrument used for this evaluation was a survey carried out after the use of the mobile application. The statistical analysis of the results obtained indicates characteristics such as the utility and perceived ease of use, positively influence students to motivate the use of mobile devices in learning a language. The results also show the great technological acceptance by students for learning and confirm that currently, mobile learning is accepted for use in education.

## Introduction

Indigenous peoples live in all regions of the world and occupy approximately 22% of Earth’s surface (Unesco, 2018). In Ecuador, 18 indigenous peoples live together with different traditions and worldviews. However, at present, due to various social, cultural and economic factors, cultural heritage of these peoples is slowly disappearing (Robles-Bykbaev et al., 2018). Kichwa is one of the 14 ancestral languages spoken in Ecuador, and is recognized in the Ecuadorian Constitution, along with Spanish, as the official language of intercultural relations (Cobo, 2015).

One of the alternatives to protect and maintain the diversity of cultural expressions is the use of information technologies, and in the case of this work, the use of mobile learning (ML). Mobile devices have generated a scenario that has an important acceptance among the current population. At present, the use of technology is gaining increasing importance and is being used as a learning tool. Access to these tools is limited by the lack of knowledge on the potential they offer and the gap between technology and daily learning activities. Fortunately, mobile devices are reversing this trend by encouraging greater integration of technology into everyday life among young people (Grasso & Roselli, 2005).

In recent years, there has been evidence of a high growth in the number of people who own a mobile device (Keengwe, Schnellert & Jonas, 2014). Cisco, in its Annual Internet Report (2018–2023) published in March 2020, predicts that more than 70% of the world’s population will have a mobile phone by 2023 (Cisco, 2020). According to the data of the Ecuadorian Institute of Statistics and Censuses (Instituto Nacional de Estadísticas y Censos, INEC), in Ecuador in 2019, 76.8% of the population had at least one smartphone activated (Albán & Sanchez, 2020).

ML aims to facilitate and improve learning strategies so that students can learn ubiquitously, that is, from anywhere and at any time using mobile devices (Madani et al., 2013; Criollo-C & Lujan-Mora, 2018a; Criollo-C & Luján-Mora, 2018b). There are several solutions that have been implemented to exploit to the maximum the use of mobile devices. Therefore, one of the topics that has had the most popularity and acceptance in recent years is ML, which has several benefits and advantages that are very helpful for students and teachers (Briz-Ponce et al., 2016; Dekhane & Johnson, 2014). Portable technologies are varied, that is why to work with ML, only smartphones and tablets are considered as the main representatives of mobile technology (Zappatore et al., 2015). These days, the high demand for handheld terminals and wireless Internet access has kept costs at bay (Cook, Pachler & Bachmair, 2011). This allows more and more people to have a mobile device of their own, thus giving rise to the first generation of fully portable information and communication technologies (Criollo-C & Lujan-Mora, 2018a; Criollo-C & Luján-Mora, 2018b).

Today’s education cannot and should not be limited to the traditional classroom; both students and teachers should have the opportunity to move, therefore fostering a collaborative education outside of the physical classroom (Criollo-C & Lujan-Mora, 2018a; Criollo-C & Luján-Mora, 2018b). Today, the proliferation of mobile applications is staggering, it is the most innovative technology in various fields including education (Briz-Ponce et al., 2016; Criollo-C, Lujan-Mora & Jaramillo-Alcazar, 2018). It is because of this that using ML can be a more natural way to engage the students in their learning process (Dingli & Seychell, 2015).

The initial hypothesis for the development of this research work was based on the question: Can the use of mobile learning applications in the learning of Kichwa language be well perceived by students? With this, it is intended that more people can access to learning this language in an easy and fun way using an educational mobile application. In addition, this proposal can be generalized to be used in the learning of other languages.

For this purpose, a mobile application was designed from several recommendations of initiatives that promote the use of mobile devices and their applications for learning (Hamada & Mitsui, 2013; Zhang, 2016).

The consulting firm International Data Corporation states that in 2019, 86.1% of users globally had a phone with an Android operating system (OS) and 13.9% with an iOS. In addition, it is expected that by 2024 this percentage will reach 86.2% for Android users and 13.8% for iOS (International Data Corporation, 2015). On that account, the research presented in this paper was initially developed only for devices that support Android OS.

The rest of the document is structured as follows. Section II shows a literature review on the acceptance of mobile technology in learning. Section III shows the design of the mobile application for learning a language. Section IV shows the methodology used for the analysis of mobile acceptance and perception of the use of mobile devices for learning a language. Section V shows the results obtained when using the mobile application. Section VI contains a discussion of the results. Section VII describes the limitations of the research conducted. Finally, the conclusions and future work are detailed in Sections VIII and IX.

### Literature review of the acceptance of mobile technology

Traditional educational methodologies such as lecture or lecture classes have been used in recent years in higher education contexts (Law, Chow & Yuen, 2005). These models promote learning by memorization and not by developing skills (reading, sharing, listening, and doing) (Bleustein-Blanchet, 2016). As a result of this, the introduction of mobile devices, together with an appropriate pedagogical design, would generate a transformation in the teaching and learning model in higher education (Kali et al., 2015). The Technology Acceptance Model (TAM) model, its extended versions, and the DeLone and McLean model (DL&ML) have been used to analyze whether quality factors and individual beliefs can generate satisfaction in students and contribute to their intention to use the mobile learning system (Briz-Ponce et al., 2016; Briz-Ponce & García-Peñalvo, 2015; Almaiah & Alismaiel, 2019). The results indicate that the quality of the system, quality of the information, quality of the service and the perceived usefulness have significant effects on student satisfaction and on their intention to use mobile learning (Almaiah & Alismaiel, 2019). These models have made it possible to show the characteristics that may influence students to adopt mobile technology (Briz-Ponce et al., 2016; Briz-Ponce & García-Peñalvo, 2015). Innovation, external influence (recommendations), perceived usefulness, perceived ease of use, self-efficacy, and positive attitude towards technology can lead students to use mobile devices in learning (Park, Nam & Cha, 2012). For example, the TAM model was used in a South Korean University to analyze the acceptance and intention of using mobile learning in students (Park, Nam & Cha, 2012). Positive attitude towards technology was found to have an effect directly proportional to intention to use and acceptance of mobile technology (Park, Nam & Cha, 2012). Meanwhile, in China it was found that both perceived utility (short and long term) and personal innovation positively influence mobile learning adoption among university students (Liu, Li & Carlsson, 2010).

Other investigations focused on the perception of students as one of the most important factors for a successful acceptance of mobile technology in the educational environment (Almaiah & Jalil, 2014; Almaiah, Jalil & Man, 2016). The results indicate the majority of students have a positive perception about the use of mobile learning, since they can access classes online using mobile devices in an easier and more useful way. They also indicate mobile devices increase learning flexibility inside and outside the classroom because students can access learning materials anytime, anywhere (Almaiah & Jalil, 2014). It is also evident that problems such as resistance to change by teachers and students, and privacy problems limit the acceptance of mobile learning (Almaiah & Al Mulhem, 2019). Furthermore, factors such as compatibility, technological readiness and culture have a negative effect on the intention to use mobile learning in specific contexts (Almaiah & Al Mulhem, 2019).

The perception of teachers regarding mobile learning is also important, which is why, a research carried out in Greece indicates the lack of professional development programs (orientation and instruction for teachers), the accessibility and usability of the devices, mobile phones, and technical requirements can hinder the decision to integrate mobile technology by teachers (Papadakis, 2018). Despite this, teachers in training reflect a positive attitude towards the use of mobile devices in education. Based on the results, as long as the factors presented above are taken into account, mobile learning could be one of the most developed educational technologies in learning environments of the future (Briz-Ponce & García-Peñalvo, 2015; Almaiah & Al Mulhem, 2019; Hamidi & Chavoshi, 2018).

### Mobile application design

**Figure 1:**
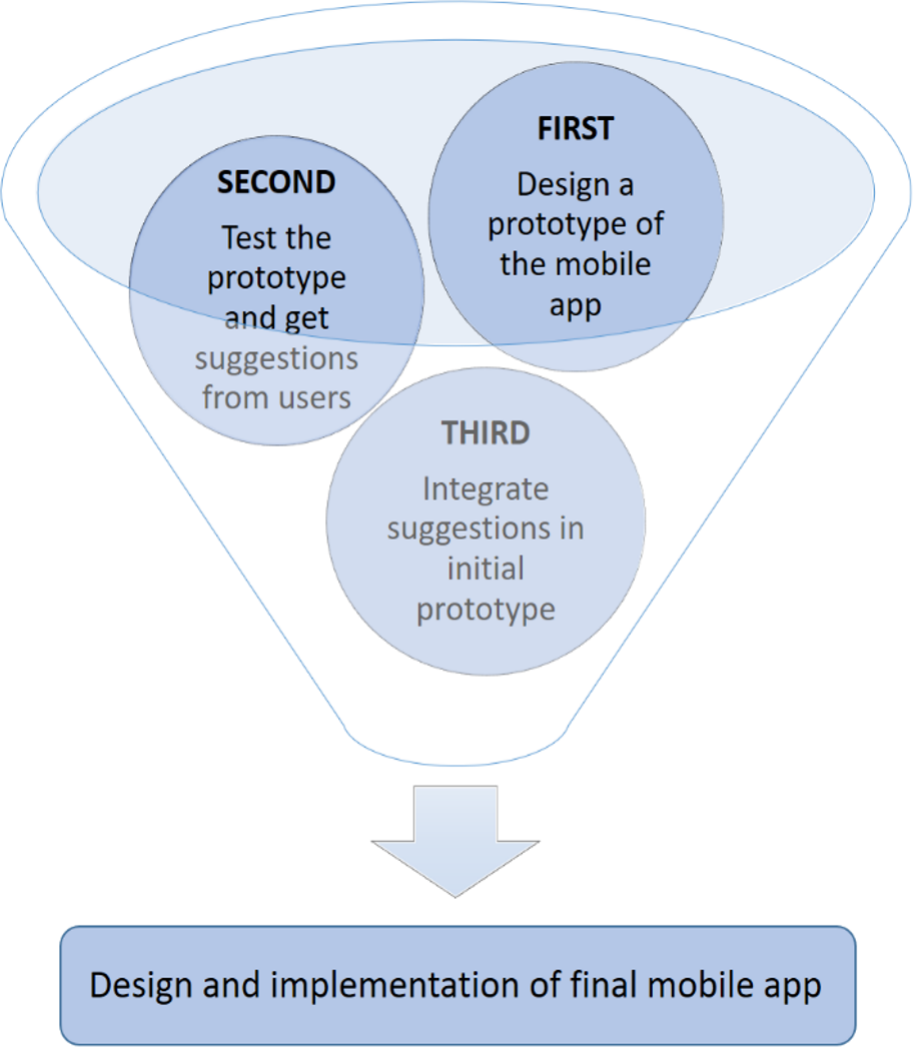
Mobile application design.

For the development of the application, it is necessary to add and implement additional factors that contribute to the achievement of the learning objective. For this, extensive research is carried out on the functional requirements for the design of mobile applications used in initiatives focused on language learning using ML. It is important to mention that the application was created to motivate learning Spanish and Kichwa languages, therefore the screenshots included in this paper contain text in the Spanish language. In each description of the images, there is a caption that provides the corresponding translation into English. Figure 1 shows the process defined for the design and creation of the mobile application.

• **First: Requirements for the design of a prototype of the mobile application**

When designing a mobile application, it is important to take into account intuition, that is, to avoid disorientation of the user due to the total number of interactions carried out Grasso & Roselli (2005). All the characteristics used for the design of the mobile application are detailed in Table 1. This table describes the requirements necessary for the mobile application to generate the intention of use by users. For example, the user’s perception regarding the work of the application must be pleasant, that is; navigation between the different game interfaces should be easy and intuitive (Ahmad et al., 2018). Each screen must be presented in a coherent way, and the functionality must resemble the menus of traditional computers interfaces (Dekhane & Johnson, 2014), etc.

**Table 1:** Requirements for the design of mobile learning.

References	Requirements	Characteristics
Ahmad et al. (2018)	**Simple and easy to use** Avoid accumulating unnecessary information on the screen Build ease of operation and multidevice compatibility	Provide ease in the game, the user should not get caught in the links of the different sections
Dekhane & Johnson (2014)	**Consistent interfaces** Design easy-to-interpret interfaces	Use known functionalities that resemble computer menus
Chuan and Guardino (2016), Taharim, Lokman & Isa (2016)	**Nice design** Use a variety of colors, fonts, and text types, should not cause confusion between links and static content. Design a friendly and attractive interface	Generate satisfaction, enthusiasm, and fun by using the different controls in the activities carried out by the application
Dekhane & Johnson (2014)	**Feedback** Comment on errors with courtesy words	Provide an understanding of mistakes made to improve task interpretation
Robles-Bykbaev et al. (2018), Grasso & Roselli (2005), Karjo & Andreani (2018), Nunkesser (2018)	**Multimedia content** Place images with adequate quality Incorporate multimedia material within the different learning modules	Generate use intent by creating multimedia interfaces that attract the user’s attention
Grasso & Roselli (2005)	**Intuition** Easy and interactive navigation through modules	Avoid user disorientation due to total number of interactions
Karjo & Andreani, (2018), Pimmer, Mateescu & Gröhbiel (2016)	**Motivation** Use emotional words at the beginning and end of the exercises	Motivate the user with kind messages while progressing through the game

The application presented in this paper has five small interactive games that were developed to reinforce the knowledge acquired. In addition, the design of the playful interfaces has the intention to make the application entertaining, easy to use, and to motivate interaction with the user. The answers collected from the users who tested the application confirm these characteristics which are defined in the following.

o **Select image**

This prototype application focuses on making use of a select group of images which are related to a text field that is located at the bottom of each image. The user-friendly interface technique utilizes an appropriate location of the text fields in conjunction with the group of available images. In addition, the images are used in association with multimedia material to capture the user’s attention and make the application entertaining.

o **Select the correct answer**

In this prototype, a question is asked where the user must select an answer from the four available options. The friendly requirement interface is used to properly locate the controls and the use of multimedia material for improved user interaction.

o **Spanish-Kichwa/Kichwa-Spanish translation**

This prototype uses one of the traditional techniques that are implemented in one of the learning methodologies, which consists in correctly translating a requested word.

o **Anagram**

The aim of this prototype is to demonstrate that, using technological tools and development platforms, it is possible to create a number of functionalities whose main focus is the interaction of the student with gamification elements.

o **Content compilation translation**

The translation system covers all the content and makes use of a table that allows to visualize the words and phrases that were reinforced throughout the learning process and exercise.

In addition, simplicity, consistency and intuition were used in the design of all prototypes. Redundancy and the use of colors and fonts that could cause confusion to the user were avoided.

• **Second: Prototype test and user suggestions**

A mobile application prototype was developed focused on learning the Kichwa language, it contained the five characteristics defined above. This project helped verify the correct operation of each of the mentioned characteristics working together.

The user has a central role in the learning process, so analysis is obviously a fundamental element for the correct design of the application. For this reason, the mobile application was tested with different people. After this assessment, the users involved in this test suggested new additional factors that complement the design established in the prototype:

Have a more interactive navigation in the application.

Avoid redundancy.

Add a limit to attempts in the exercises.

Define clear instructions or suggestions on what to do in the exercises.

Provide independence and adaptability of content in different modes of interaction.

• **Third: integrate the suggestions in the initial prototype**

The mobile application prototype was designed using the requirements detailed in Table 1. Furthermore, the feedback from individuals who use the prototype, helped include additional features that were excluded in the initial design. The minimum requirements that can be implemented in the design of a mobile application focused on learning a language are defined.

• **Design and implementation of the final mobile application**

**Figure 2:**
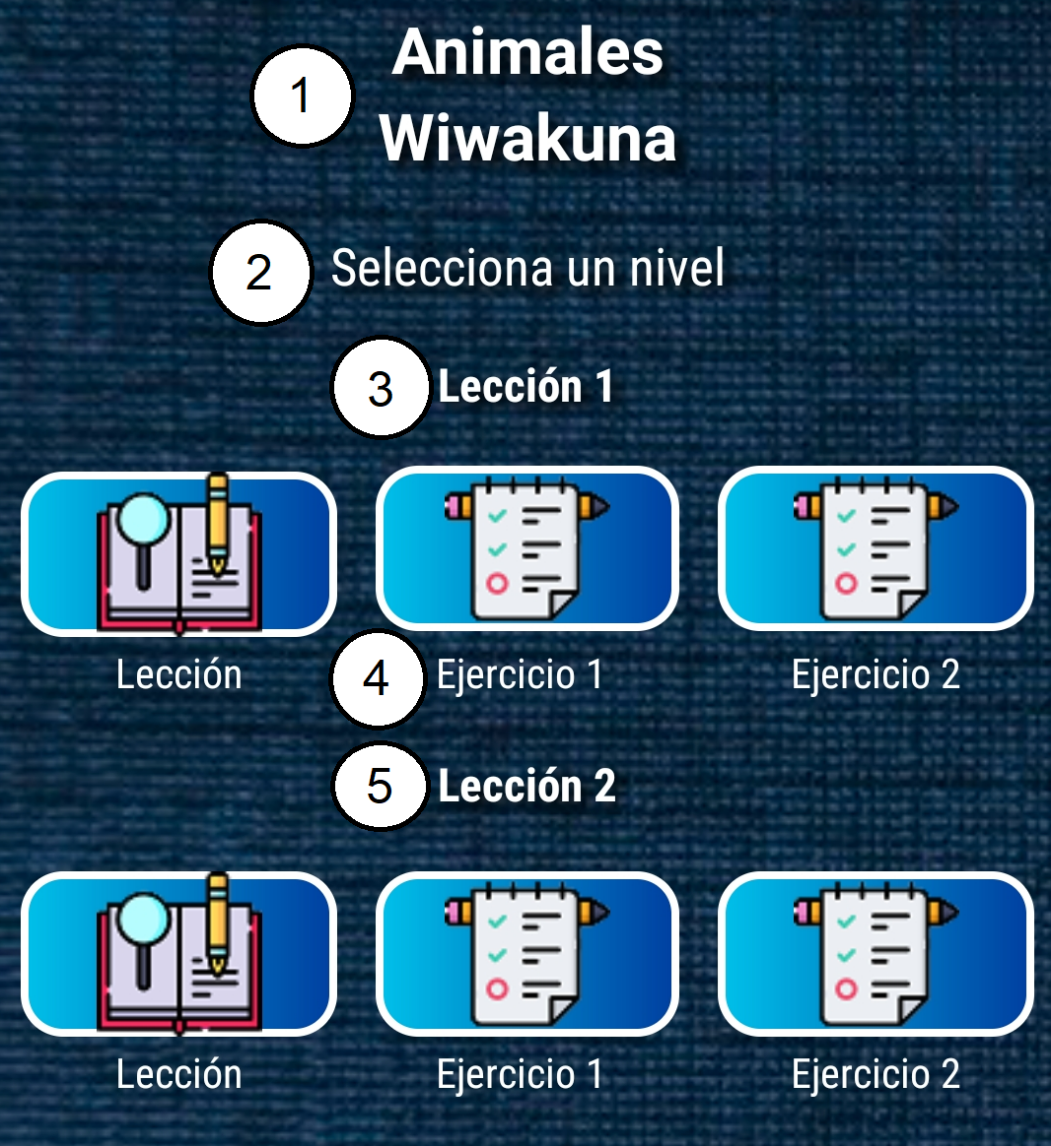
Level lesson interface. 1. Animals/Wiwakuna; 2. Select a level; 3. Lesson 1; 4. Exercise 1; 5. Lesson 2.

**Figure 3:**
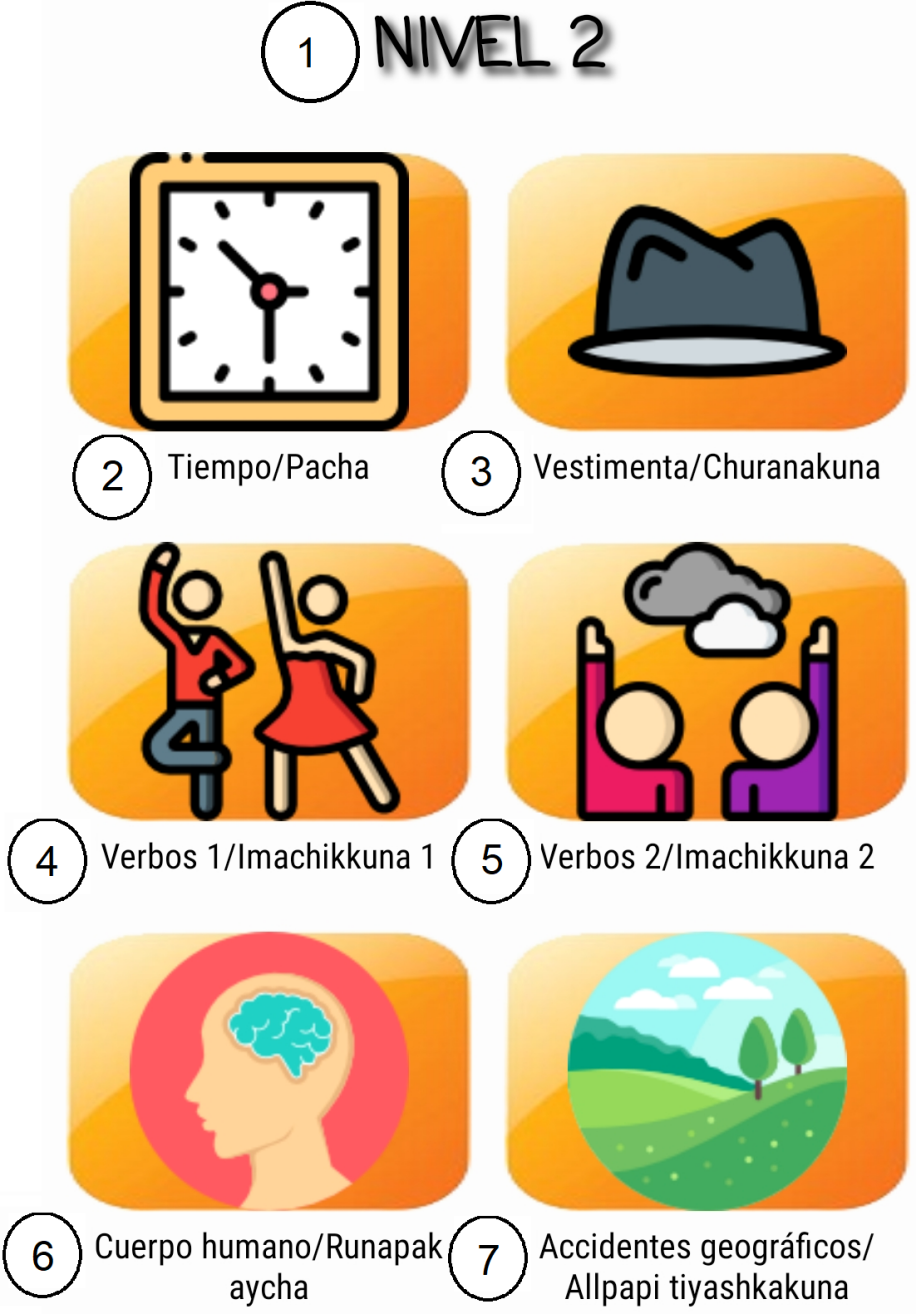
Application main screen. 1. Level 2; 2. Time/Pacha; 3. Cloths/Churanakuna; 4. Verbs 1/Imachikkuna 1; 5. Verbs 2/Imachikkuna 2; 6. Body/Runapakaycha; 7. Terrain topography/Allpapi tiyashkakuna.

**Figure 4:**
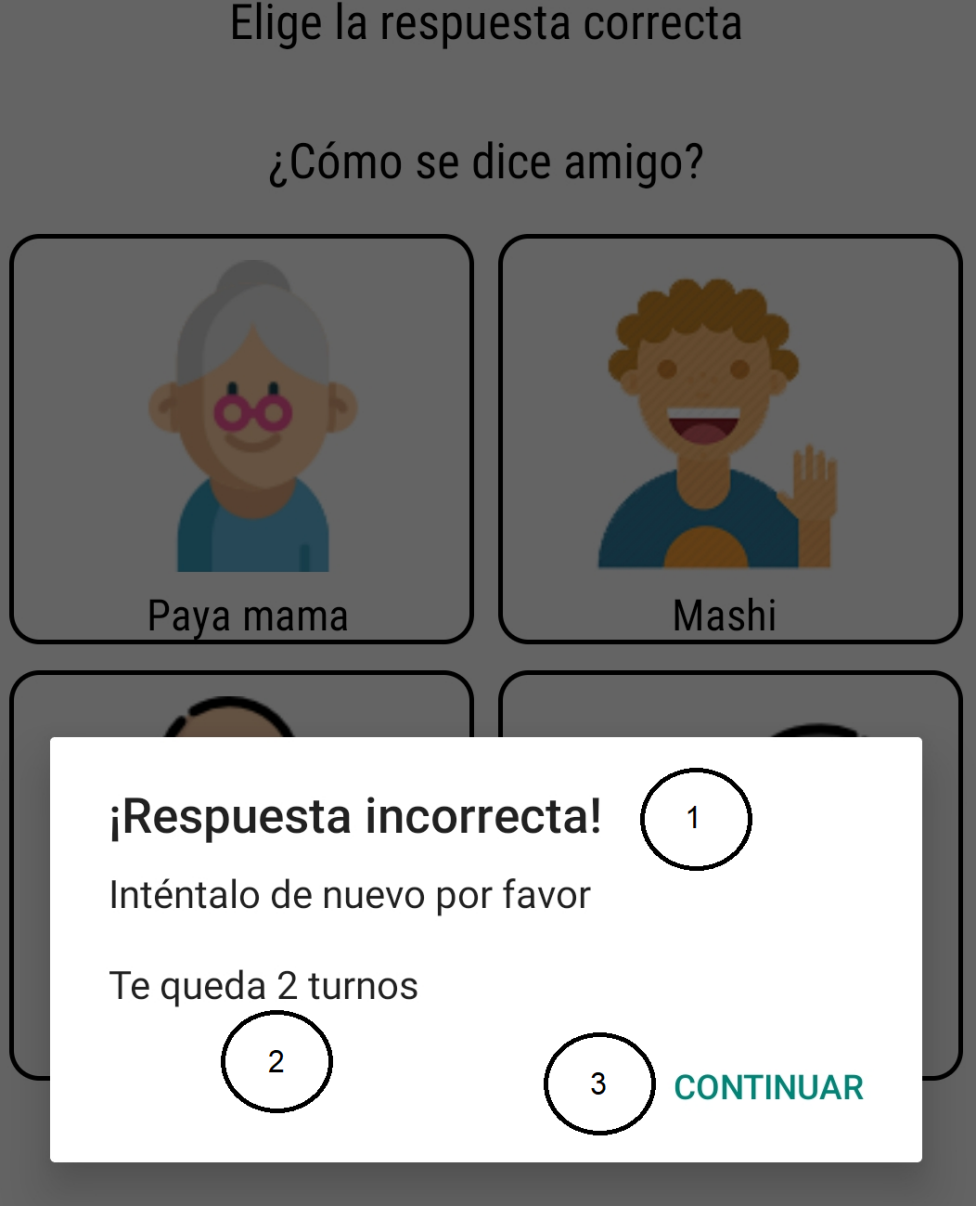
Use of Courtesy words. 1. Incorrect answer! Try again please; 2. You have 2 attempts left 3. Continue.

**Figure 5:**
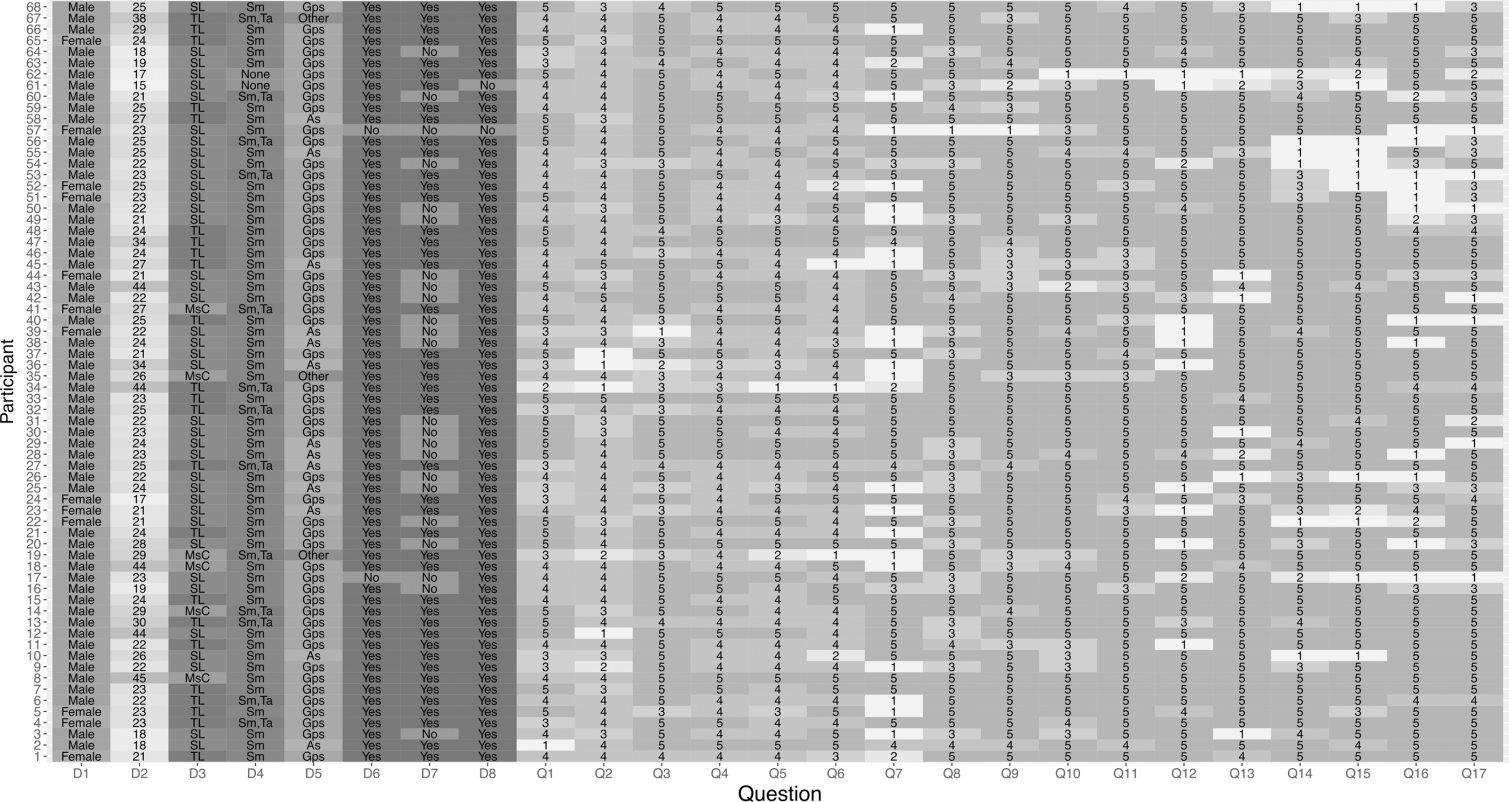
Answers (categorical and numerical) for demographic data, acceptance of mobile learning and UTAUT* model. *UTAUT = Unified theory of acceptance and use of technology.

The ultimate goal is to provide the user with a pleasant and motivational experience when making use of the application. Several of these characteristics can be seen in Figs. 2, 3, 4 and 5. The main functionalities for the design and implementation of the final mobile application were:

User friendly interface.

Simplicity, consistency and intuition.

Avoid redundancy.

Have a more interactive navigation in the application.

Use colors, fonts and types of text to avoid confusion.

Use of images with good screen resolution.

Use of courtesy and emotional words.

Incorporation of interactive and multimedia material.

Immediate feedback.

Define clear instructions or suggestions on what to do in the exercises.

Independence and adaptability of content in different modes of interaction.

The app has six types of main interfaces to carry out this work. They perform different tasks and are implemented throughout the operation and execution of the application. From these interfaces, other secondary windows will be created that handles the same methodology but with certain changes in the location of the controls, actions they perform, and content. For example, as can be seen in Figs. 4 and 5, the mobile application implemented design methodologies that allow better organization of display and access to content. Both Figs. 2 and 3 show nice, simple, and easy-to-use interfaces.

In addition, the interfaces that were created to improve the interaction of users with the application were the following: user input interface, main menu interface, level interface, level lesson interface, and exercise interface of the level. After the primary interfaces are established, the secondary gaming interfaces are also created. These were developed as interactive games that help reinforce the user’s knowledge and use the functionalities detailed above.

Examples of these secondary interfaces (playful) can be found in Fig. 4. This show the features necessary to create an application that captures the user’s attention. Figure 4 shows a multiple choice question and each of its answers contains images that help to choose the correct answer, in addition, the mobile application shows the errors that can be made and the limit of attempts allowed.

## Materials & Methods

The Dean of the Faculty of Engineering and Applied Sciences, University of the Americas granted approval to carry out this anonymous survey. We applied Design Science Research (DSR) to verify the hypothesis of this work (Hevner et al., 2004). This approach was used as it allows the development of valid and reliable knowledge to design solutions, solve problems, create changes or improve existing solutions (Van Aken, 2004). The DSR process includes six activities: (1) identification of the problem and justification of the value of a solution, (2) definition of objectives to create a solution, (3) design and development of the solution, (4) demonstration using the solution to solve the problem; (5) evaluation of the solution, comparing the objectives and the real results observed from its use, and (6) communication of the problem, the solution, its usefulness and effectiveness for other research and future work. Activities 1 and 2 have been detailed in the summary and introduction of this work. The remaining four activities can be evidenced in the methodology, presentation, and discussion of results that we present in the following.

A. **Instruments**

We started by having each participant sign an informed consent. The instrument used for this research was a survey of 25 questions distributed in three parts. The first part consisted of 8 questions that collected demographic information categorically. The second part consisted of 7 questions that collected information on the perception of users for the use of mobile devices in learning a language. The third part consisted of 10 questions that were designed according to the Unified Theory of Acceptance and Use of Technology (UTAUT) (Nitsche, 2013). The second and third parts used a five-point Likert scale to quantify the numerical responses. This UTAUT model is based on the Technology Acceptance Model (TAM) that uses the perceived utility (PU) and the perceived ease of use (PEU) as external factors (Davis, 1985). In addition, it incorporates three additional elements: Social Influence (SI), Perceived Entertainment (PE), and Facilitating Conditions (FC) (Sánchez-Prieto, Olmos-Migueláñez & García-Peñalvo, 2017). The 25 questions used in the three surveys are detailed below.

•: **Sociodemographic data (categorical responses)** D1. Gender D2. Age D3. Education level D4. Mobile device type D5. On which virtual platform do you download your applications? (Google Play Store, Apple App Store) D6. Do you currently have your own mobile device? D7. Do you have a mobile data plan? D8. Do you have Internet access at home?
•: **Perception of the use of mobile learning by users (numerical responses on a LIKERT scale of 1 –5)** Q1. How important is the use of mobile devices for academic learning? Q2. Do you use or have you used a mobile application to learn a language? Q3. Would you like to learn a new language using the mobile device? Q4. Do you think that the mobile device is a tool that supports the learning of academic subjects? Q5. Do you think that with the help of mobile devices you can improve a person’s academic performance? Q6. Do you think that in the future, with the advancement of technology, mobile devices will be essential in class hours? Q7. Do you agree that mobile devices are used as learning tools during class hours?
•: **Questions related to UTAUT (numerical responses likert scale 1–5)** Q8. (PU1) Do you think that the mobile device is a tool that supports language learning? Q9. (PU2) Do you think that if you use a mobile device you will learn a language faster? Q10. (PEU1) Is it easy to use a mobile device to learn a language? Q11. (PEU2) Is it easy to learn how to use a mobile device and use it in education? Q12. (SI1) Did a teacher or person in authority promote the use of mobile devices to learn a language? Q13. (SI2) Does a close relative think that you can learn a language using mobile devices? Q14. (FC1) Is it easy to download documentation and applications to learn a language? Q15. (FC2) Is it easy to use applications to learn a language? Q16. (PE1) Is it fun to use the mobile device to learn a language? Q17. (PE2) Do I like to learn a language much more if I do it using a mobile device?
B.: **Experimental protocol**

Each participant received instruction on how to use the mobile application and responded to the first two parts of the survey. Participants were able to ask their questions after using the mobile prototype and made their recommendations. The experimentation lasted about 25 min per each participant. Subsequently, the participants answered a final questionnaire with 10 questions related to the UTAUT. Display tests were conducted on 10 different types of smartphones and tablets. The app can be said to work and display properly on various mobile devices. The people who took the surveys provided invaluable information regarding the use of mobile devices along with their individual and group preferences. This information allows us having a real idea of the use of ML in current education, specifically to learn a language. In addition, this information is useful for teachers and educational institutions that want to include ML to innovate traditional methodologies and thus adequately meet current learning challenges. This information also makes it possible to know what the challenges and problems that could arise when deploying this technology in the teaching-learning process may be.

## Results

### Participants and demographic data

The population under study were the students of a Kichwa language learning center in the city of Otavalo (Ecuador) in 2019. In Ecuador there are not many places for the teaching of the Kichwa language, most are located in the province of Imbabura (Ecuador), which is why the sample of participants is the most representative at the national level with respect to people who want and can learn the Kichwa language. Figure 5 shows the results of the three parts of the survey in categorical and numerical values. The categorical responses (D1-D8) of the first questionnaire (S1) indicate that the number of people who answered it was 68, the demographic data indicate that 19% were female and 81% male. The age range was from 15 to 45 years. Regarding the educational level of the participants, 57% were high school graduates (second level, SL), 34% were university graduates (third level, TL), and 9% had a graduate or master’s degree (MSc). In addition, from the survey responses, it was evident that 76% of those surveyed had a mobile device with Android and downloaded their applications from the Google Play Store (Gps). 19% had a device with iOS and downloaded their applications from the Apple App Store (As). Another interesting fact was that 19% of those surveyed owned two devices, a smartphone (sm) and a tablet (ta), and 78% owned only one sm. Only 3% did not have their own mobile device. Of the participants who have their own mobile device, only 68.18% have a mobile data plan for Internet access. Ecuador has managed to reduce the digital divide, and this is reflected in the residential Internet access of the participants: 97% of them have Internet access at home, only 2 participants do not own their own mobile devices, nor do they own residential Internet access.

### Variance analysis on the acceptance of mobile learning

Figure 5 also contains the numerical responses of the two surveys: acceptance of ML (SAML) and the UTAUT model. These values have a range of responses with the following weighting: I strongly disagree = 1; I disagree = 2; I am so so = 3; I agree = 4; I strongly agree = 5. In this figure, according to the predominant colors in the answers, a good acceptance can be seen for all the questions (Q1–Q17). These results will be analyzed in more detail in the following figures.

**Figure 6:**
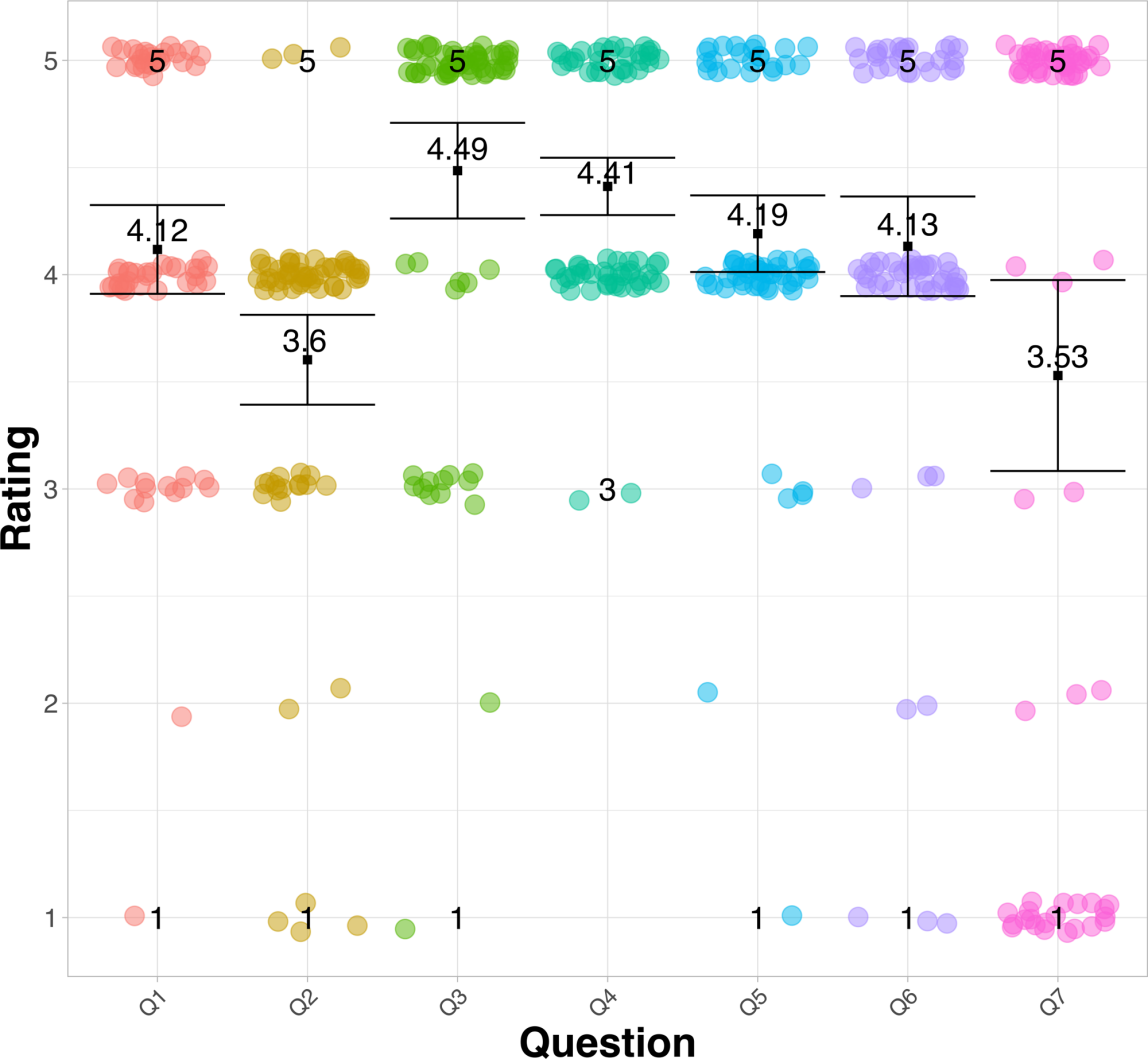
Diagram of the average responses to the acceptance questions of mobile learning.

Figure 6 shows a strip diagram; these represent the average response of the seven questions about the acceptance of the ML. The results obtained indicate that there is a positive assessment by the participants regarding the use and acceptance of mobile devices for use in education. Tables 2 and 3 show the values of the mean, median, and standard deviation of the responses about the perception of the use of ML and the UTAUT technology acceptance model, respectively. In Table 2 can be observed that the perceptions of the use of mobile devices in ’most cases’ have an average higher than 4.12, and the standard deviation was less than 0.96. In Fig. 5, question Q3 and Q4 graphically show that participants would like to use ML as they believe it is useful in the learning process of a language. It can also be observed that question Q2 and Q7 are the least accepted by the participants. Although the results suggest that Q2 and Q7 have less influence on the acceptance of the ML, this does not mean that there is opposition from the participants, it only reflects the value of the mean, which is lower than the rest of the questions. The answers to question Q2 and Q7, as indicated in Table 2, are the only ones with a mean lower than 4. question Q7 is the only question that has 21 answers in 1 (totally disagree), no other question has this feature therefore, it has a standard deviation of 1.84 and a mean of 3.53 as indicated in Table 2. This means that ML technology is not fully accepted for use in the educational process. Moreover, 35% of the participants do not agree that mobile devices are used as learning tools during class hours The low acceptance of the use of ML technology in the classroom may respond to a possible distraction, which may cause students to deviate from the objective in the teaching and learning process (Unesco, 1990).

**Table 2:** Characteristics of mobile learning acceptance survey.

Survey	Question	Answers	µ	M	*σ*
Perception of the use of mobile learning	Q1	68	4.12	4.00	0.86
	Q2	68	3.60	4.00	0.87
	Q3	68	4.49	5.00	0.92
	Q4	68	4.41	4.00	0.55
	Q5	68	4.19	4.00	0.74
	Q6	68	4.13	4.00	0.96
	Q7	68	3.53	5.00	1.84

**Notes.**

*μ* Mean M Median *σ* Standard deviation

**Figure 7:**
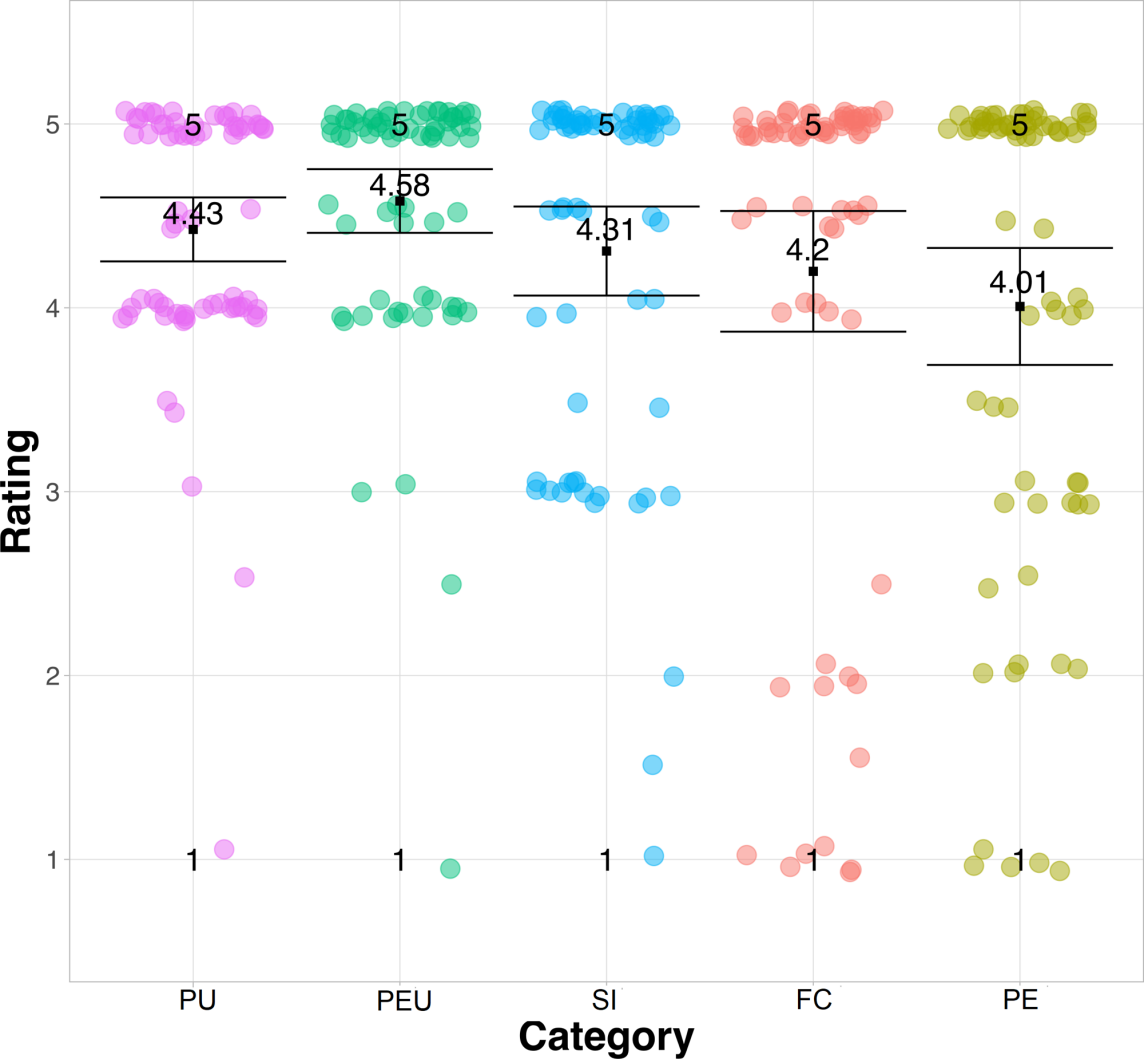
Diagram of the average responses to the questions of the UTAUT model.

Question Q2, as can be seen in Fig. 6, has a high density of responses in 3 (I am so so) and 4 (I agree). This means that participants have little experience in using a mobile application to learn a language. Despite these statistics, it can be concluded that the participants have a very good acceptance of ML technology, as indicated visually in Figs. 6 and 7.

As the analysis was carried out with a 95% confidence level, the results indicate that there are no differences between the groups of question Q1, Q3, Q4, Q5, Q6. Furthermore, the average of the results of these questions is higher than 4.12. That is, most users would like to use their mobile device to learn, and they believe it is a support tool for improving academic performance and will be used more frequently in the future.

### Variance analysis on the UTAUT model

The results of the 10 questions of the UTAUT model are shown in a strip diagram with the average response for the five categories in Fig. 7. According to the values corresponding to the mean, median, and standard deviation shown in Table 3, it can state that there are no significant differences between the five categories of the UTAUT model. This means that PU, PEU, SI, FC, and PE have a positive influence on technological acceptance among participants.

**Table 3:** Characteristics of UTAUT model survey.

UTAUT model	Question	Answers	µ	M	*σ*
Perceived utility (PU)	Q8	68	4.32	5.00	0.98
	Q9	68	4.53	5.00	0.91
Easy to use (PEU)	Q10	68	4.53	5.00	0.91
	Q11	68	4.63	5.00	0.81
Social influence (SI)	Q12	68	4.21	5.00	1.48
	Q13	68	4.41	5.00	1.26
Facilitating conditions (FC)	Q14	68	4.25	5.00	1.30
	Q15	68	4.15	5.00	1.53
Perceived entertainment (PE)	Q16	68	3.93	5.00	1.62
	Q17	68	4.09	5.00	1.37

**Notes.**

*μ* Mean M Median *σ* Standard deviation

This can also be seen in the results obtained, in most cases, there is a mean greater than 4.09 and a standard deviation less than 1.53. In addition, due to the level of confidence used for this analysis (95%), it can be seen graphically in Fig. 7 that the difference between the groups of the questions of the UTAUT model is almost zero. Despite this, it is important to mention that the mean values of PU =4.43 and PEU =4.58 (Table 3) indicate that these two items are the ones that most positively influence the intention to use ML to learn a language. Although the results suggest that the SI, FC, and PE have less influence for the use of ML, this does not mean that there is an opposition from the participants, it only reflects the value of the mean, which is less than the rest of the external factors (SI =4.30, FC =4.20, PEU =4.0).

### Correlation analysis

**Figure 8:**
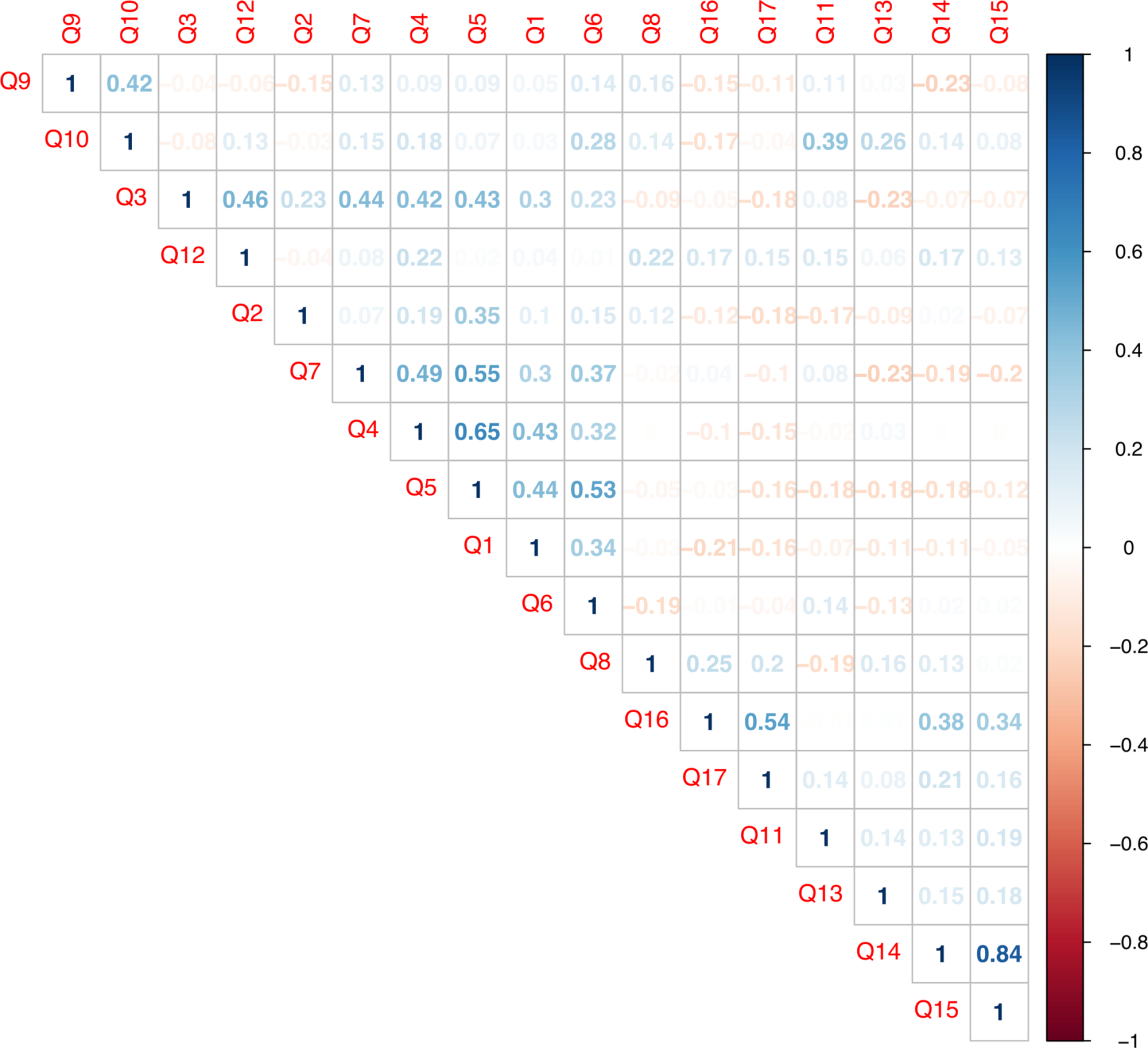
Correlation diagram between acceptance questions of mobile learning and the UTAUT model.

Figure 8 shows the correlation only between numerical questions. The correlation diagram was made with questions from the questionnaires on the acceptance of ML (Q1–Q7) and the UTAUT model (Q8–Q17). The relationship between the questions in these two surveys is marked positively in blue and negatively in red. It can be observed that there is a greater correlation between questions of the UTAUT model (Q14–Q15; Q16–Q17). This means that the participants responded positively and very similarly to the questions about facilitating conditions and perceived entertainment. That is, ML as a technology has a positive acceptance and these two characteristics are the main reason among users.

Other important correlation values (greater than 0.5) are found in the questions about the acceptance of mobile learning (Q4–Q5; Q5–Q6; Q5–Q7). This means that the participants responded in a similar way to all these questions, and they agree on their importance. It can be said that the participants support the use of mobile devices in the educational field. They strongly believe that mobile devices are academic support and can improve academic performance. Furthermore, they support the idea that mobile devices will be present in the future of education. Although there is a significant number of people (35%) who oppose the use of this technology in the classroom, the rest of the respondents have no doubts in stating the opposite categorically.

**Figure 9:**
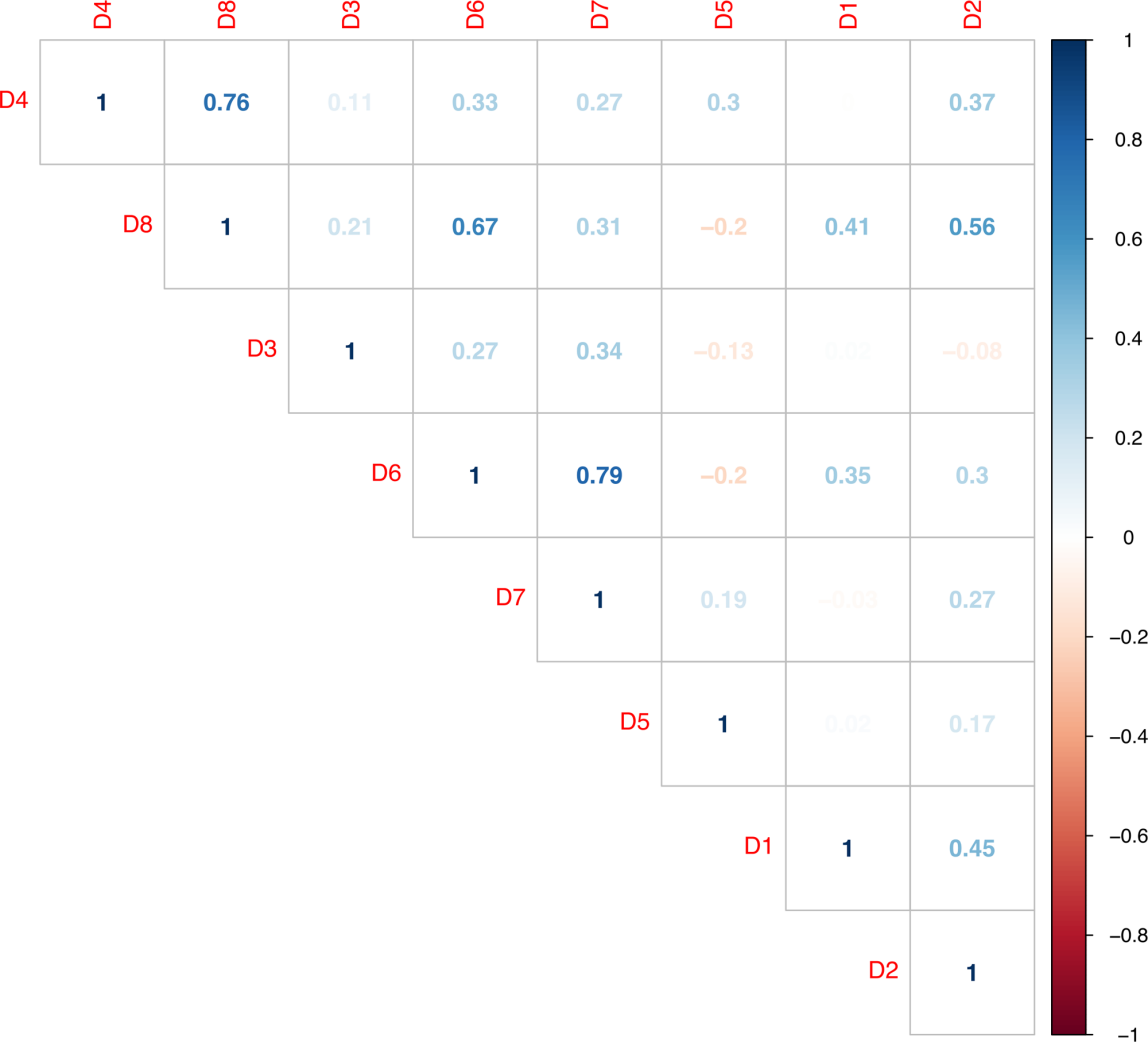
Correlation diagram between demographic questions.

The correlation between the categorical questions of the demographic data of the first questionnaire can be seen in Fig. 9. The interpretation is similar to Fig. 8, the highest relationship is represented by the blue color and the lowest by the red color. The graph shows a correlation between several questions, for example, the highest correlation is between question D6 - D7. This analysis is performed by including the clustering data defined for this study. This correlation (D6 - D7) indicates the following: the participants who own their own mobile device also have a mobile data plan for Internet access through their mobile devices. Another important connection is question D4 - D8 in relation to all the other questions, with exception of gender and type of mobile OS respectively. This relation indicates that older people, who have a home and mobile Internet access, have a higher level of education and own two of their own devices (smartphone and tablet).

**Figure 10:**
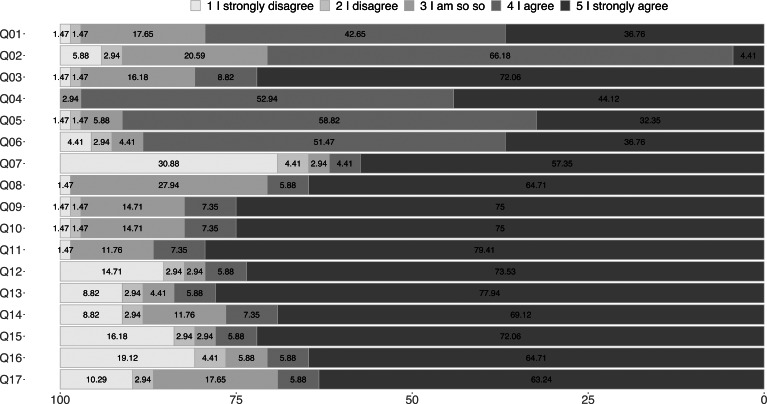
General acceptance and UTAUT model regarding mobile learning.

The other relationships will be defined in the following analysis. Despite the fact some responses show little acceptance of the use of ML, this resistance is low compared to the acceptance of this technology as an aid in the educational process and learning a language. The support and acceptance of mobile technologies as a strategic partner in education can also be observed. It is evident that the participants believe in the importance of ML and believe that these devices should be used to improve student performance and support learning. The summary of the responses on the Likert scale regarding the use and acceptance of ML can be seen in Fig. 10. Here it is evidenced that mobile devices are increasingly accepted in education. People would like to include their mobile devices in their learning because of the ease with which it can be used for this purpose.

### Cluster’s analysis

**Figure 11:**
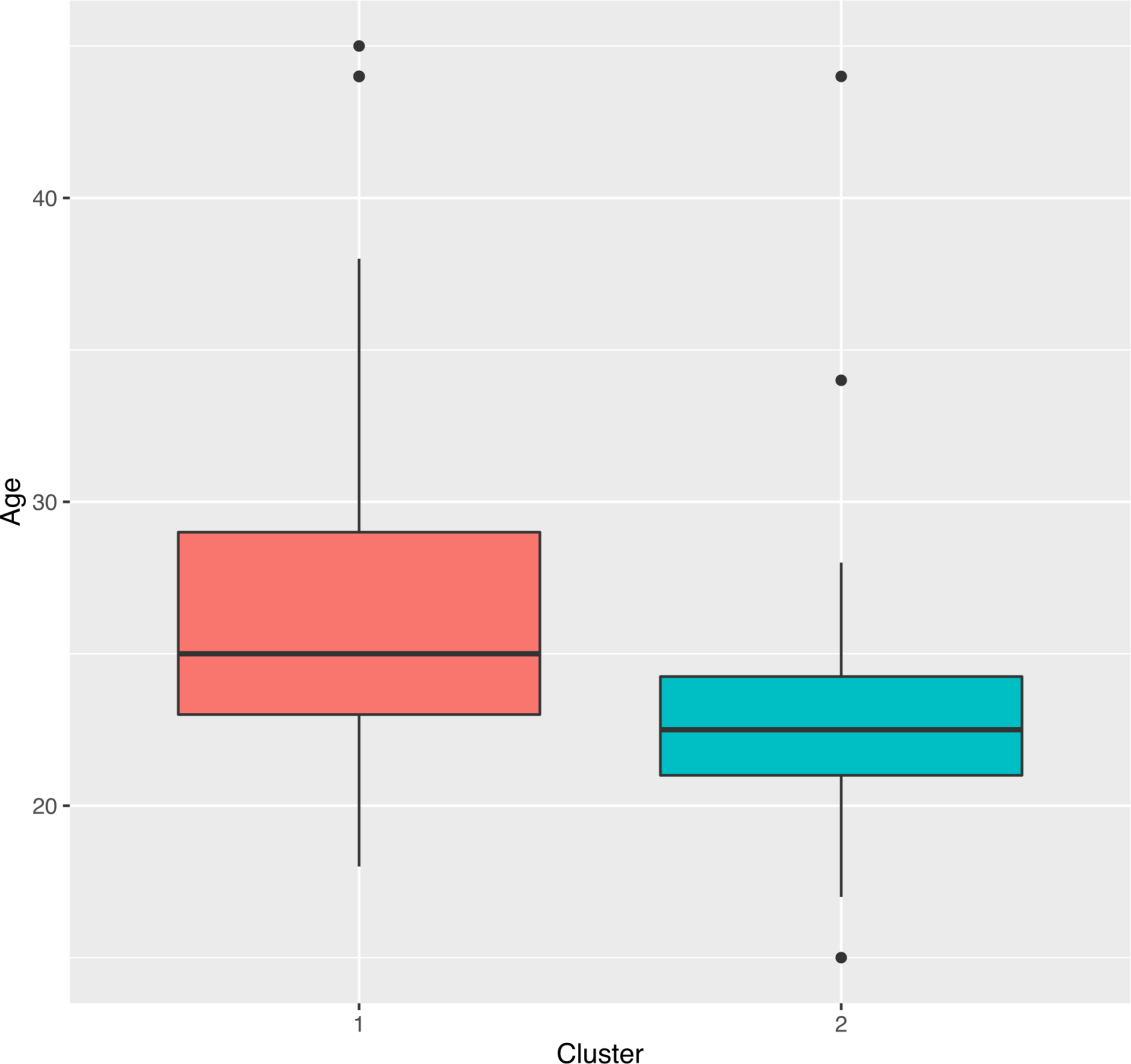
Optimal number of found clusters.

**Figure 12:**
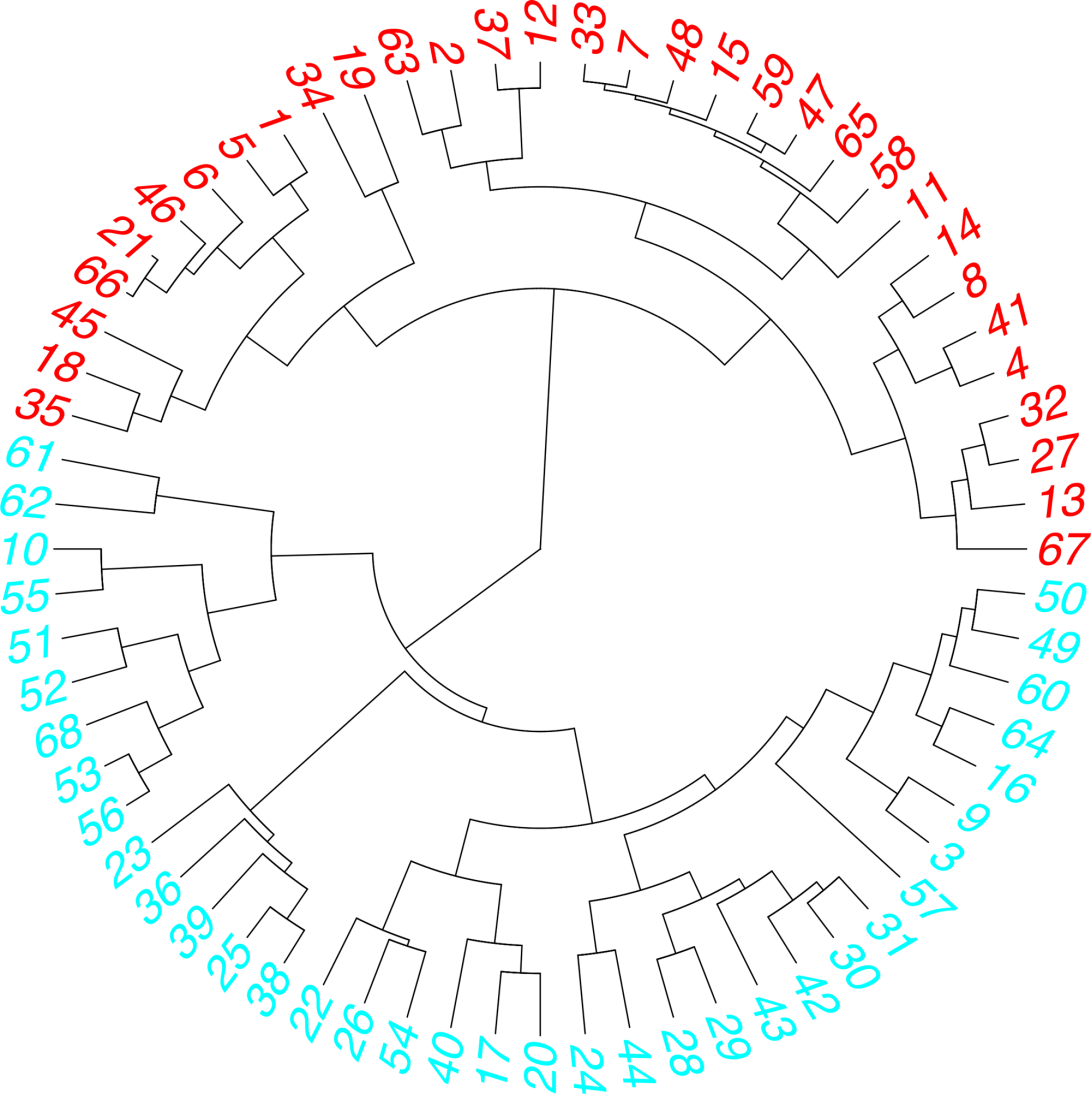
Dendrogram of data concentered in two clusters.

A cluster analysis based on the similarity of the responses was also performed (Beckstead, 2002). This analysis is responsible for clustering a set of individuals, in such a way that people in the same cluster have very similar responses to each other. The similarity between observations is defined using some distance measures based on the correlation of survey questions and answers. The method used for this analysis was hierarchical clustering, by the Gower distances between all responses (categorical and numerical) (Beckstead, 2002; Gower, 1971; Murtagh, 2002). With these data, a distance tree was constructed that was used to find the discrete clustering based on the similarity of their responses. Figure 11 shows the optimal number of clusters found using the hierarchical method (C1 and C2). Figure 12 is a dendrogram that contains the clustering of the responses of the 68 participants concentrated in the two groups. In Fig. 11 we can be seen the two-clustering taking as an example the age of the participants and their respective cluster. For example, cluster 1 has the oldest participants. In Fig. 12 it can be seen that 2 is the appropriate number of clusters for this analysis (there are two predominant colors).

The exploratory analysis of these two clusters can generate new information regarding the acceptance of the ML and the characteristics of the UTAUT model. The objective of the cluster analysis (C1 and C2) is to find new information that may be relevant to test the hypothesis defined at the beginning of the investigation (Beckstead, 2002).

## Discussion

The results grouped by cluster are shown in Tables 4 and 5; they indicate that there is a favorable tendency for the majority of participants (C1 and C2) to use ML as support in education. The main reasons are the mobile device serves as a learning aid and people would like to learn using it. On the other hand, the cluster C2 indicates perceived entertainment has the lowest acceptance for the respondents.

Table 4 shows the responses of the participants in cluster C1 regarding the two numerical surveys about the use of ML and the UTAUT model. This first clusters contains 38% of women and 49% of men, that is, 47% of the total number of participants. Furthermore, in C1, 50% of the participants are over 25 years of age (Fig. 11).

Table 5 shows the responses of the participants in cluster C2 regarding the two numerical surveys about the use and acceptance of ML. It contains 62% women and 51% men, that is, 53% of the total number of participants. In C2, 75% of the participants were under 25 years of age (Fig. 11). Also 71% of those who have access to mobile Internet, in addition, there is 77% of those who own 2 mobile devices (Sm, Ta). Finally, 48% of those surveyed who have access to the home Internet are grouped here.

For the cluster C2, has the youngest users, it contains all the respondents who do not own a mobile device (3%). In addition, it contains 90% of college graduates. It also contains 58.5% of participants who own a single device (smartphone) and 51.5% of participants who do not have a mobile device of their own. Here are all the participants who do not have access to home and mobile Internet. Tables 3 and 4 show a summary of the cluster analysis for the numerical responses on the Likert scale. We can see that the participants in cluster C1 support both the use and the acceptance of ML. Their responses are grouped on the most favorable Likert scale (3, 4, and 5). Cluster C2 participants support the use and acceptance of ML. However, their responses reflect that social influence, facilitating conditions and perceived entertainment have less impact on the acceptance of ML. The clustering allows defining the common interest of the participants and can guide us in the search for answers on the research hypotheses. This work addresses the possibility of generating motivation in learning the Kichwa language.

**Table 4:** Likert scale responses by clustering C1.

Category	Question	Likert scale				
		1	2	3	4	5
Perception of the use of mobile learning	Q1	1	1	5	13	12
	Q2	3	1	4	22	2
	Q3	0	0	6	5	21
	Q4	0	0	1	16	15
	Q5	1	1	1	20	9
	Q6	3	0	1	15	13
	Q7	9	3	0	3	17
Perceived utility (PU)	Q8	0	0	3	3	26
	Q9	0	0	8	5	19
Easy to use (PEU)	Q10	0	0	4	2	26
	Q11	0	0	3	2	27
Social influence (SI)	Q12	1	0	1	1	29
	Q13	0	0	0	3	29
Facilitating conditions (FC)	Q14	0	0	0	1	31
	Q15	0	0	2	2	28
Perceived entertainment (PE)	Q16	0	0	0	3	29
	Q17	0	0	2	2	28

**Notes.**

I strongly disagree = 1, I disagree = 2, I am so so = 3, I agree = 4, I strongly agree = 5

According to the results observed throughout the research, it can be stated the participants have a positive attitude towards ML. They also claim that this technology could be used to learn a language. That is, most of the respondents would like to learn the Kichwa language with the support of the designed application. In addition, they support the use of mobile applications together with ML as a strategic partner in the educational process.

**Table 5:** Likert Scale responses by clustering C2.

Category	Question	Likert scale				
		1	2	3	4	5
Perception of the use of mobile learning	Q1	0	0	7	16	13
	Q2	1	1	10	23	1
	Q3	1	1	5	1	28
	Q4	0	0	1	20	15
	Q5	0	0	3	20	13
	Q6	0	2	2	20	12
	Q7	12	0	2	0	22
Perceived utility (PU)	Q8	1	0	16	1	18
	Q9	1	1	2	0	32
Easy to use (PEU)	Q10	1	1	6	3	25
	Q11	1	0	5	3	27
Social influence (SI)	Q12	9	2	1	3	21
	Q13	6	2	3	1	24
Facilitating conditions (FC)	Q14	6	2	8	4	16
	Q15	11	2	0	2	21
Perceived entertainment (PE)	Q16	13	3	4	1	15
	Q17	7	2	10	2	15

**Notes.**

I strongly disagree = 1, I disagree = 2, I am so so = 3, I agree = 4, I strongly agree = 5.

Although this study shows little evidence of opposition to ML, the majority of respondents agree the correct use of these devices would improve academic performance. In addition, they believe mobile devices are useful and easy to use, so they would contribute greatly to the current educational model. The results obtained confirmed the hypothesis raised, the technology, through the mobile application “Otavalo Rimay”, can be well perceived by students for learning the Kichwa language.

Based on the results presented, this study provides valuable information on the key factors to consider in teaching-centric mobile applications. It is evidenced that students’ observations (perceived usefulness and perceived ease of use) promote satisfaction and the intention to use mobile technology. These findings strengthen the results of other research, for example in Briz-Ponce et al. (2016), Briz-Ponce & García-Peñalvo (2015) and Almaiah & Alismaiel (2019) it is indicated that perceived usefulness has significant effects on student satisfaction and their intention to use mobile learning. Other work indicates that perceived ease of use also indirectly affects the behavioral intention to use mobile learning (Almaiah, Jalil & Man, 2016). The results of this research contribute scientific evidence to the technological acceptance for learning a language, also confirming that, currently, mobile learning is widely accepted for use in education.

## Limitations

This research has a very important limitation that has to do with the number of participants involved in this initiative. The sample of participants is small (68), this is due to the fact that there are not many Kichwa language teaching centers in Ecuador, most of these centers are located in the province of Imbabura (Ecuador). Therefore, the number of participants should be increased not only from one teaching center but from several and in different provinces within Ecuador. Other participants from countries with Kichwa-speaking communities such as Bolivia, Peru, Colombia, Chile, and the northern Argentina could also be involved.

Another limitation of the research is that an analysis showing learning outcomes using a control group was not performed. This is partly due to the low number of participants who took part in the study. In addition, this study was conducted during the COVID-19 pandemic, which was a challenge to its development. The absence of a covert question and answer method, in which the participant is not aware that he is being part of an experiment and is being observed, can be considered a limitation of this work, the use of this method can avoid alterations in the configuration of the experiment. Another limitation of the work was not considering in the questions of the questionnaire the negative aspects or impacts that the users could perceive as a result of their experience when using the mobile application. Finally, another limitation was the absence of an “open question” so that the participants could communicate any opinion about the application and the experiment carried out.

## Conclusions

Technological advances make it possible for technologies such as ML to be used to prevent the disappearance of a language like Kichwa, which is of incalculable cultural value. Despite the initiatives for the use of ML as support in educational issues, the results show that there is some resistance to the adoption of this new technology. For example, the results indicate that for some participants, facilitating conditions, perceived entertainment, and social influence were less attractive for the adoption of ML. This is because these devices can become distracting and not allow the proper development of learning. All this due to the use of social networks, online games, and collaboration tools. However, it is possible to implement measures to avoid this inconvenience. As on a computer, on a mobile device, it is possible to install applications to temporarily block access to other applications, block notifications, and thus increase user concentration. The use of technology can only facilitate learning, but the real learning outcome will appear only if there is a specific requirement that motivates this learning. Mobile learning should be used as a way to motivate learning in new generations of digital natives through innovative educational methodologies. Current generations grew up with social media, mobile devices, and Internet access. This means that their way of receiving information is linked to technologies. That is why educators and educational institutions must promote the effective use of this technology. The “Otavalo Rimay” application is available in the Google Play Store for free download and use. The work carried out seeks to encourage the learning of this language and that is why any person or educational institution can use it freely.

## Future Work

This project opens up new lines of research, which could include improvements in the design of the mobile application to help motivate the learning of Kichwa. For example, it would be useful to include Kichwa phonetics by placing the sound of words and phrases through multimedia material. Without a doubt, this would help the correct pronunciation of the language and would contribute positively to its learning. One of the current technological trends in teaching is the use of educational games. Likewise, it is proposed to migrate the mobile application to other platforms, for example iOS, and implement accessibility so that it can be used by people with disabilities. It is proposed as a scope of this project that the research methodology used includes the opinions of experts and people who know the language. Their experience and support would be a complement that would improve the results of this research. Besides, a future work should focus on analyzing the effectiveness of the mobile application using a control group and an evaluation. The results of this evaluation can show whether or not there was an influence for the improvement of learning with the use of the application. Also, it is important, to include in the questionnaire a question about; whether users would recommend the use of this mobile application to other people. It is also important to include in the questionnaire questions the negative aspects or impacts that users could perceive as a result of their experience when using mobile devices. As well as a question that evidence and analyzes the probability that the participant postpones learning due to the use of mobile devices. The answers to these questions would indicate the degree of satisfaction, besides, will generate a list of disadvantages, and problems to be addressed in new research papers. Other researchers are encouraged to implement more gamification processes, such as collaborative use of the application so that multiple users can use the interactive part simultaneously with several mobile devices.

Future work should focus on the design of appropriate pedagogical methodologies for the use of mobile technologies. Finally, in a future initiative, a descriptive and open question should be included in the questionnaire to collect the impressions that the participants may have regarding the use of the application.

##  Supplemental Information

10.7717/peerj-cs.550/supp-1 Supplemental Information 1 Original Mobile Application Research survey (in Spanish)

10.7717/peerj-cs.550/supp-2 Supplemental Information 2 Research Survey Mobile Application

10.7717/peerj-cs.550/supp-3 Supplemental Information 3 Data Survey

## Additional Information and Declarations

### Data Availability

The following information was supplied regarding data availability: The Supplemental File (Data Survey) contains all the responses to the survey carried out on the participants of the research carried out.
