# Supplementary material for: Exploring the technological acceptance of a mobile learning tool used in the teaching of an indigenous language
Source: PeerJ Comput Sci. 2021 Jun 3;7:e550. doi: 10.7717/peerj-cs.550 (PMC8189028; doi:10.7717/peerj-cs.550)
Supplement: Supplemental Information 1 [file peerj-cs-07-550-s001.pdf]

Encuesta de Investigación sobre aplicación móvil

D1. Genero
Femenino
masculino

D2. Edad

D3. Nivel de Educación
Segundo nivel
Tercer nivel
Master or PhD

D4. Tipo de dispositivo móvil utilizado
Teléfono inteligente
Tableta

D5. ¿De qué plataforma virtual descarga sus aplicaciones?
Google Play Store
Apple App Store
Otra

D6. ¿Tiene actualmente su propio dispositivo móvil?
Si
No

D7. ¿Tienes un plan de datos móviles?
Si
No

D8. ¿Tiene acceso a Internet en casa?
Si
No

Para la siguiente parte, revise lo siguiente: (Estoy totalmente en desacuerdo = 1; No estoy de acuerdo = 2; Estoy tan = 3; Estoy de acuerdo = 4; Estoy totalmente de acuerdo = 5)

Q1. ¿Qué importancia tiene el uso de dispositivos móviles para el aprendizaje académico?
1 2 3 4 5

Q2. ¿Utiliza o ha utilizado una aplicación móvil para aprender un idioma?
1 2 3 4 5

Q3. ¿Le gustaría aprender un nuevo idioma usando el dispositivo móvil?
1 2 3 4 5

Q4. ¿Cree que el dispositivo móvil es una herramienta que apoya el aprendizaje de materias académicas?
1 2 3 4 5

Q5. ¿Cree que con la ayuda de los dispositivos móviles se puede mejorar el rendimiento académico de una persona?
1 2 3 4 5

Q6. ¿Cree que, en el futuro, con el avance de la tecnología, los dispositivos móviles serán imprescindibles en el horario de clases?
1 2 3 4 5

Q7. ¿Está de acuerdo en que los dispositivos móviles se utilizan como herramientas de aprendizaje durante las horas de clase?
1 2 3 4 5

Q8. (PU1) ¿Cree que el dispositivo móvil es una herramienta que apoya el aprendizaje de idiomas?
1 2 3 4 5

Q9. (PU2) ¿Cree que si usas un dispositivo móvil aprenderás un idioma más rápido?
1 2 3 4 5

Q10. (PEU1) ¿Es fácil utilizar un dispositivo móvil para aprender un idioma?
1 2 3 4 5

Q11. (PEU2) ¿Es fácil aprender a utilizar un dispositivo móvil y utilizarlo en la educación?
1 2 3 4 5

Q12. (SI1) ¿Un profesor o una persona con autoridad promovió el uso de dispositivos móviles para aprender un idioma?
1 2 3 4 5

Q13. (SI2) ¿Un familiar cercano piensa que se puede aprender un idioma usando dispositivos móviles?
1 2 3 4 5

Q14. (FC1) ¿Es fácil descargar documentación y aplicaciones para aprender un idioma?
1 2 3 4 5

Q15. (FC2) ¿Es fácil usar aplicaciones para aprender un idioma?
1 2 3 4 5

Q16. (PE1) ¿Es divertido usar el dispositivo móvil para aprender un idioma?
1 2 3 4 5

Q17. (PE2) ¿Me gusta mucho más aprender un idioma si lo hago usando un dispositivo móvil?
1 2 3 4 5
