# Supplementary material for: Exploring the technological acceptance of a mobile learning tool used in the teaching of an indigenous language
Source: PeerJ Comput Sci. 2021 Jun 3;7:e550. doi: 10.7717/peerj-cs.550 (PMC8189028; doi:10.7717/peerj-cs.550)
Supplement: Supplemental Information 2 [file peerj-cs-07-550-s002.pdf]

Research Survey Mobile Application

D1. Gender
  Female
  male

D2. Age

D3. Education level
  Second Level
  Third Level
  Master or PhD

D4. Mobile device type
  Smartphone
  Tablet

D5. On which virtual platform do you download your applications?
  Google Play Store
  Apple App Store
  other

D6. Do you currently have your own mobile device?
  Yes, I do
  No, I do not

D7. Do you have a mobile data plan?
  Yes, I do
  No, I do not

D8. Do you have Internet access at home?
  Yes, I do
  No, I do not

For the next part check the following: (I strongly disagree = 1; I disagree = 2; I am so so = 3; I agree = 4; I strongly agree = 5)

Q1. How important is the use of mobile devices for academic learning?

1 2 3 4 5

Q2. Do you use or have you used a mobile application to learn a language?

1 2 3 4 5

Q3. Would you like to learn a new language using the mobile device?

1 2 3 4 5

Q4. Do you think that the mobile device is a tool that supports the learning of academic subjects?

1 2 3 4 5

Q5. Do you think that with the help of mobile devices you can improve a person's academic performance?

1 2 3 4 5

Q6. Do you think that in the future, with the advancement of technology, mobile devices will be essential in class hours?

1 2 3 4 5

Q7. Do you agree that mobile devices are used as learning tools during class hours?

1 2 3 4 5

Q8. (PU1) Do you think that the mobile device is a tool that supports language learning?

1 2 3 4 5

Q9. (PU2) Do you think that if you use a mobile device you will learn a language faster?

1 2 3 4 5

Q10. (PEU1) Is it easy to use a mobile device to learn a language?

1 2 3 4 5

Q11. (PEU2) Is it easy to learn how to use a mobile device and use it in education?

1 2 3 4 5

Q12. (SI1) Did a teacher or person in authority promote the use of mobile devices to learn a language?

1 2 3 4 5

Q13. (SI2) Does a close relative think that you can learn a language using mobile devices?

1 2 3 4 5

Q14. (FC1) Is it easy to download documentation and applications to learn a language?

1 2 3 4 5

Q15. (FC2) Is it easy to use applications to learn a language?

1 2 3 4 5

Q16. (PE1) Is it fun to use the mobile device to learn a language?

1 2 3 4 5

Q17. (PE2) Do I like to learn a language much more if I do it using a mobile device?

1 2 3 4 5
